# Control of Intra-Thymic αβ T Cell Selection and Maturation by H3K27 Methylation and Demethylation

**DOI:** 10.3389/fimmu.2019.00688

**Published:** 2019-04-03

**Authors:** Rémy Bosselut

**Affiliations:** Laboratory of Immune Cell Biology, Center for Cancer Research, National Cancer Institute, National Institutes of Health, Bethesda, MD, United States

**Keywords:** thymus, T cell development, histone methylation, histone demethyalse, polycomb, H3K27 methylation

## Abstract

In addition to transcription factor binding, the dynamics of DNA modifications (methylation) and chromatin structure are essential contributors to the control of transcription in eukaryotes. Research in the past few years has emphasized the importance of histone H3 methylation at lysine 27 for lineage specific gene repression, demonstrated that deposition of this mark at specific genes is subject to differentiation-induced changes during development, and identified enzymatic activities, methyl transferases and demethylases, that control these changes. The present review discusses the importance of these mechanisms during intrathymic αβ T cell selection and late differentiation.

## Introduction: Chromatin and Control of Gene Expression

Pioneering studies in prokaryotes have led to the paradigm that adjusting gene transcription in response to environmental signals involves transcription factors, proteins that bind specific DNA sequences (cis-regulatory elements) close to the transcription start site. Such binding promotes DNA-templated RNA synthesis by the RNA polymerase. The same paradigm governs the control of transcription in eukaryotic cells, with added layers of complexity at virtually every step, including the multiplicity of RNA polymerases, the functional overlap among trans-acting factors, and the unsuspected promiscuity of transcription factors with cis-regulatory elements. Typical genes are controlled by multiple, often tissue-specific cis-regulatory elements, potentially distantly located relative to the transcription start site. Such elements are bound by transcription factor assemblies which themselves typically recruit cofactor complexes that mediate their action on the polymerase complex.

In addition, eukaryotes use two important layers of controls of gene expression, DNA methylation and chromatin dynamics. Eukaryotic DNA is methylated on cytosines located upstream of a guanine, and stretches of such palindromic CpG dinucleotides (called CpG islands) are frequently found in cis-regulatory elements. Their methylation status is inversely correlated with gene expression (1). The impact of CpG methylation is not limited to transcriptional silencing, as it affects transcription factor binding, positively or negatively depending on the transcription factor and target sequence (2). Additionally, eukaryotic DNA is packaged into nucleosomes and higher-order nucleosome-based structures referred to as chromatin, in which DNA is tightly associated with histones, thereby restraining its accessibility to transcription factors or to the polymerase machinery. Such packaging is dynamic and subject to two sets of modifications. First, “chromatin remodeling,” performed by energy-dependent enzymatic complexes, changes the position of nucleosomes over DNA; this process is essential to “open” specific regulatory sequences for transcription factor binding or polymerase recruitment (3). Second, histone molecules themselves are subject to covalent modifications, including acetylation, methylation, and ubiquitination (4). Many of these modifications occur on specific amino-acid residues within the amino-terminal “tail” of histone molecules, that is not tightly associated with DNA. Through their combinatorial effect, these modifications constitute a high-order “code,” that has a broad impact on chromatin structure and gene expression (5, 6). Covalent modifications are “written” (added) or “erased” (by catalytic removal) by specific enzymatic complexes, and recruit “reader” protein complexes that affect transcription.

Specific histone modifications are associated with specific gene expression states or regulatory regions (4). Acetylation of histone H3 on lysines 9 or 27 (H3K27Ac or H3K9Ac) is preferentially found at enhancers or promoters of expressed genes. Similarly, methylation of H3 lysine 4 is associated with active enhancers (H3K4 mono- or di-methylation) or found at the promoter of actively transcribed genes (H3K4Me3). In contrast, H3 K9 methylation, and in particular tri-methylation, is associated with heterochromatin formation. This review focuses on the methylation of H3 lysine 27 (H3K27Me3), which has attracted much interest because of its association with lineage-specific gene repression and because its impact on transcription is in large part mediated through its interactions with Polycomb Repressive Complexes (PRC), which were initially identified as controllers of homeotic gene expression in Drosophila (7, 8).

There is compelling evidence that changes in H3 K27 methylation are not simply associated with gene expression status, but have a causative role in setting gene transcription levels (9–11). However, it has been difficult to quantify the actual contribution of this mechanism because chromatin modifications and sequence-specific transcription factors serve cooperatively to control transcription, and because these mechanisms mutually affect each other with multiple examples of interactions between transcription factors and H3K27Me3 writer, eraser or reader complexes (12, 13). Additionally, the genetic tools available for such studies, i.e., inactivation of chromatin modifiers, methyl-transferases and demethylases for H3K27Me3, by definition have a broad impact on the transcriptome, complicating mechanistic studies. The present review will discuss how these mechanisms control H3K27Me3 homeostasis in the thymus and contribute to the development of αβ T cells.

### αβ T Cell Development

#### Early Stages

T cell development in the thymus is a multi-step process combining cell proliferation, differentiation and survival-selection events (14). As a result, it has attracted interest not only because of the essential role of T cells in immune responses, but also because it is one of the few developmental processes that is amenable to both genetic and functional studies after the completion of embryonic development. Two main lineages of T cells can be separated based on the composition of their heterodimeric antigenic receptor: αβ and γδ T cells, respectively expressing TCRα and TCRβ, or TCRγ and TCRδ chains. All T cells derive from bone marrow precursors, and their development can be divided into three schematic steps: (i) T cell lineage commitment, common to both αβ and γδ lineages (15–17), (ii) antigen receptor gene rearrangement and commitment to either of the two main T cell lineages (αβ vs. γδ) (18, 19), and (iii) selection-maturation of αβ- and γδ-committed T cells. This review will focus on the selection and maturation of αβ lineage T cells (20), a process involving acquisition of long-term survival, choice of either of the two main lineages of αβ T cell, defined by the expression of CD4 and CD8 surface molecules (14), and intrathymic migration events that culminate in the egress of mature thymocytes to the blood circulation and their entry in secondary lymphoid organs.

#### Conventional T Cell Differentiation From Early αβ Lineage Precursors

**Figure 1:**
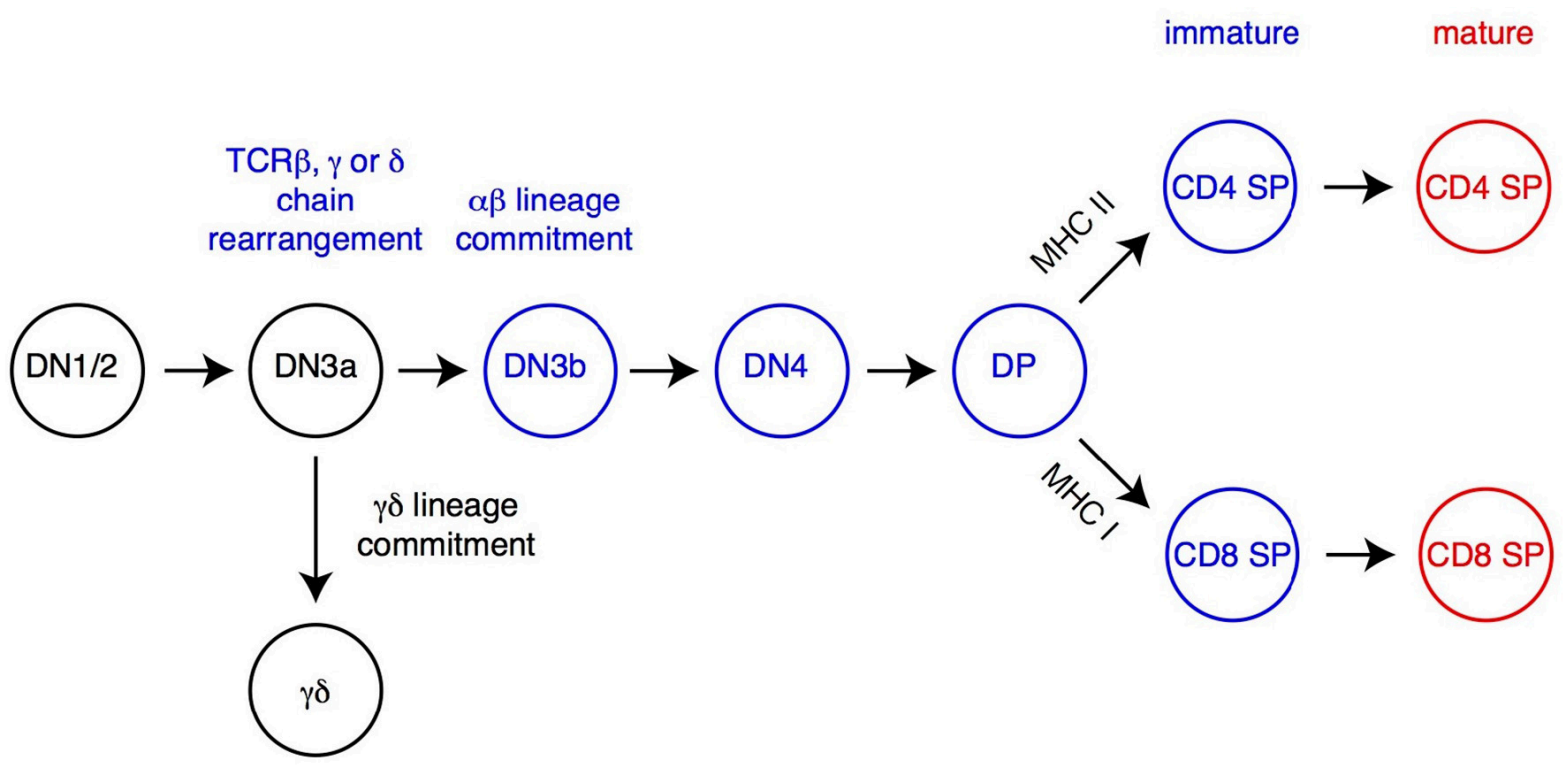
Overview of T cell development. The most immature T cell precursors (CD4^−^CD8^−^DN thymocytes), CD4^+^CD8^+^ DP and CD4 and CD8 SP thymocytes are depicted. Relevant DN thymocyte subsets (defined on expression of CD25 and CD44) are shown (14, 21). Commitment to either αβ or γδ lineage occurs after successful rearrangement, at the early DN3 stage (DN3a, characterized by low expression of surface markers CD27 and CD28), of the corresponding TCR chains (TCRβ for αβ lineage cells, TCRγ and TCRδ for γδ lineage cells). DP thymocytes signaled by MHC-I or MHC-II-associated peptides undergo positive selection, differentiate into the CD8 or CD4 lineage (respectively) and complete their maturation before leaving the thymus. Cells signaled by high-affinity ligands are either deleted (negative selection, not depicted) or directed toward specific fates, including iNK T cells (Figure 2) or regulatory T cells (Treg, not depicted).

The earliest αβ lineage-committed thymocytes have successfully rearranged one of their TCRβ-encoding genes and express neither CD4 nor CD8 coreceptors (“double-negative” [DN] thymocytes) (Figure 1). After they have up-regulated both molecules (and are thus called “double-positive” [DP]), these cells rearrange their TCRα genes, allowing the surface expression of TCRαβ complexes which “probe” the set of MHC peptide complexes expressed by thymic epithelial cells (22). In the absence of productive MHC-peptide interactions (and therefore signaling though their TCR), these short-lived cells undergo programmed cell death in the thymic cortex within 3 days of their generation (23). In contrast, thymocytes that express an αβ TCR with appropriate affinity for MHC peptide complexes are rescued from cell death, a process referred to as positive selection (24–27); positive selection is closely associated (and possibly mechanistically linked) to the termination of TCRα gene rearrangement and changes in chemokine receptor expression that will eventually lead DP thymocytes from the cortex to the thymic medulla (28). Of note, cells with high avidity for MHC peptide complexes are either targeted for activation-induced cell death (“negative selection” by deletion) or diverted to alternate developmental fates, most notably differentiation into regulatory T cells with suppressive activity (29–31). Although the latter processes are critical for immune tolerance, they have not been shown to be affected by H3K27 methylation and will not be further discussed below.

Positively selected DP thymocytes differentiate into either CD4- or CD8-lineage T cells, defined by the cessation of either CD8 or CD4 expression and accompanied by “pre-programming” for helper vs. cytotoxic functions, respectively (32–34) (Figure 1). The “choice” of lineage is determined by the cell's MHC specificity, so that thymocytes that recognize MHC-II bound peptides become CD4^+^ T cells, whereas those recognizing MHC-I-bound peptides become CD8^+^ T cells (35). This process involves multiple transcription factors, including two with lineage specific expression, the zinc finger molecule Thpok in CD4^+^ thymocytes and Runx3 in CD8^+^ thymocytes (36–39). Following their CD4-CD8 differentiation, differentiating αβ lineage thymocytes undergo terminal maturation, including expression of surface receptors enabling their migration to secondary lymphoid organs after thymus exit, and of S1pr1, a sphingosine phosphate receptor needed for thymic egress (40, 41).

The differentiation of DP thymocytes into mature T cells involves extensive changes in gene expression (42), accompanied by modifications of the chromatin landscape (43–45). Unlike in many other differentiation processes, αβ lineage thymocytes do not divide during their intrathymic differentiation into mature T cells (23, 46). Thus, changes to the chromatin landscape cannot be mediated by “dilution” of chromatin marks but must be implemented by active mechanisms that remove or add chromatin marks on relevant genes.

#### Innate-Like αβ T Cells Undergo Effector Differentiation in the Thymus

**Figure 2:**
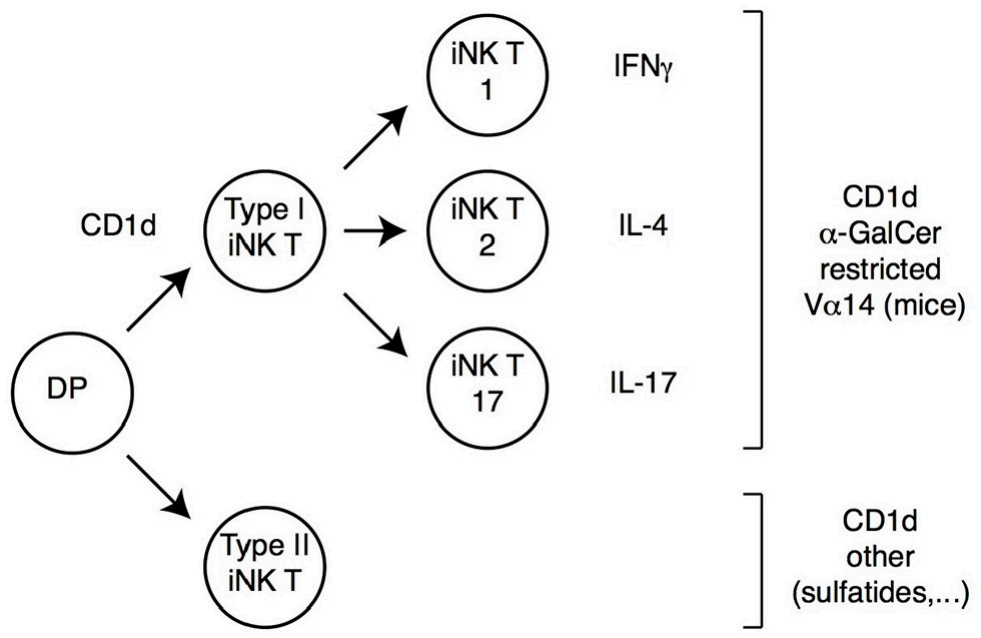
iNK T cell subsets. DP thymocytes signaled by CD1d-bound lipids differentiate into iNK T cells. Most iNK T cells (Type I iNK T cells) express a Vα14 invariant TCRα chain (and exhibit a reduced TCRβ chain diversity), and recognize CD1d-bound α-Galactosyl-Ceramide (αGalCer). These cells undergo functional differentiation in the thymus (requiring the transcription factor PLZF) into IFNγ, IL-4 or IL-17-expressing effector fates (therefore referred to as iNK T1, T2, or T17, respectively). A smaller subset of CD1d-signaled cells (Type II iNK T cells) does not carry the prototypical Vα14 chain and does not recognize αGalCer; these cells also undergo functional differentiation, although specific cytokine expression patterns are not as extensively characterized as for Type I iNK T cells.

In addition to classical MHC-I or MHC-II molecules, DP thymocytes can be signaled by MHC-like molecules and differentiate into “innate-like” or “non-conventional” αβ T cells, which acquire effector functions during their intrathymic differentiation. By far the best characterized among these cells are invariant natural killer (iNK) T cells, which recognize lipids bound to CD1d molecules (47–49). In mice, most iNK T cells express a TCR including a specific Vα14 Jα18 TCRα chain paired to a restricted set of TCRVβ chains; such type I iNK T cells react with CD1d-bound α-galactosyl ceramide (αGalCer), and can be identified through their binding to a tetramerized version of this complex (Figure 2). In contrast, type II iNK T cells, while also CD1d-restricted, do not bind CD1d-αGalCer, and do not express the canonical Vα14 Jα18 chain (50, 51).

Regardless of the ligand they recognize, iNK T cells differ from conventional T cells in multiple respects (Figure 2). They are selected in the thymus by CD1d molecules expressed by DP thymocytes (unlike conventional thymocytes which are selected by MHC-I or MHC-II molecules expressed by the thymic epithelium), and their development requires homotypic interaction between SLAM-family receptors expressed on both the CD1d-presenting cell and the CD1d-signaled differentiating iNK thymocyte (47). As a result of these signals, iNK T precursors up-regulate the zinc finger transcription factor PLZF, and undergo intrathymic proliferation and effector differentiation (52–55). The resulting mature iNK thymocytes acquire differentiation programs and cytokine production pattern typical of Th1, Th2 or Th17 effector T cells; they express the corresponding fate-specific transcription factors (T-bet, Gata3 and RORγt, respectively) and are thus called NKT1, NKT2, and NKT17 cells (54); note that this “functional” classification is unrelated to the aforementioned distinction between type I and type II iNK T cells, which refers to ligand specificity. The acquisition of effector functions by iNK T cells in the thymus contrasts with the vast majority of conventional thymocytes, which do not acquire effector properties during their development and leave the thymus as “naïve” T cells. Importantly, analyses in recombinant mice have shown that PLZF is both necessary and sufficient for the implementation of the NK T effector program, and the control of PLZF expression and function is therefore a critical factor in iNK T differentiation. Last, most iNK T cells colonize effector sites in tissues rather than secondary lymphoid organs, most prominently the liver and gut mucosa, where they contribute to the recognition of CD1d-bound microbial metabolites (48).

### Enzymatic Activities Carrying H3K27 Methylation and Demethylation

#### H3 K27 Methylation and Methyl Transferases

Nucleosomes carrying trimethylated H3K27 are preferentially located at and near promoters of silent genes (7, 56–58). There is evidence that H3K27Me3 actually contributes to transcriptional repression, mostly by recruiting Polycomb-repressive complex 1 (PRC1), which is considered as the main H3K27Me3 “reader.” Recruitment is mediated by direct binding of H3K27Me3 to PRC1 Cbx subunits (8, 12, 59), although recent studies have highlighted the role of long non-coding RNAs in modulating these interactions and PRC1 functions (60–62). When recruited to chromatin, other PRC1 subunits repress transcription, notably by promoting histone H2A ubiquitination (63). Additionally, the methylation of H3 K27 prevents its acetylation and thereby indirectly contributes to transcriptional repression. Polycomb-repressive complexes 2 (PRC2) “write” the H3K27Me3 modification, through their catalytic components Ezh1 or Ezh2 methyl transferases (7, 64). Both Ezh2 and components of PRC1 are critical at multiple stages of immune cell development and responses, highlighting the importance of H3K27 methylation for cell homeostasis and differentiation (65–72).

#### H3K27Me3 Demethylases

Conversely, H3K27Me3 can be “erased” by catalytic demethylation (into di- and monomethyl forms) by Jmjd3 and Utx demethylases. These enzymes belong to a large family defined by the presence of a complex catalytic domain, called JmjC (73–77). Their demethylase activity requires oxygen and α-ketoglutarate, and is therefore controlled by the cell metabolic status. The protein sequences of Jmjd3 and Utx are largely unrelated outside of their JmjC domain, suggesting that these molecules have unique, and potentially non-redundant, demethylase-independent activities. *In vitro* analyses suggest a strict correspondence between Jmjd3 and Utx catalytic activities and H3K27Me3 demethylation. That is, both molecules are highly specific for H3K27Me3, relative to other methylated histone residues (78–83), whereas most other JmjC-based demethylases have no significant *in vitro* activity on H3K27Me3.

Importantly, both H3K27 methyl-transferases and H3K27Me3 demethylases have histone-independent activities. Ezh2 methylates non-histone substrates, including cytosolic factors controlling actin polymerization and TCR signaling (66, 72). It was also reported to methylate and promote the degradation of the transcription factor PLZF needed for iNK T cell differentiation (84, 85). Jmjd3 and Utx have demethylase-independent activities and are notably part of KTM2 complexes (also called MLL), which are found at the promoter of active genes (86) and include H3 Lysine 4 histone methyl transferases (hence the KTM name). Both Jmjd3 and Utx were reported to associate with specific (and distinct) KTM2 complexes (87, 88), in which they may serve a structural (scaffold-like) role, or promote association with transcriptional regulators. In addition, Jmjd3 and Utx interact with Brg1-based chromatin remodeling complexes (89), which displace nucleosomes over the DNA (3) and have notably been implicated in the control of *Cd4* and *Cd8* expression and T cell development (90, 91). For Jmjd3, this association is independent of its demethylase activity (89) and has been reported to be important for the function of the transcription factor T-bet during the differentiation of activated CD4^+^ T cells into Th1 effectors (92).

#### H3K27Me3 Erasers: Do They Matter?

Early studies of H3K27Me3 homeostasis raised a puzzling paradox. They found that disruption of Polycomb genes (writers or readers) has a strong impact on cell differentiation and function in multiple experimental systems, including in ES cells and embryonic development, tumor development, and early hematopoiesis (93–96). This is in line with experiments in Drosophila and analyses of tumor-specific mutations in pediatric glioblastoma, which indicate that H3K27 trimethylation causes, rather than results from, transcriptional repression (10, 11). In contrast, and unexpectedly, disrupting H3K27Me3 erasing, by impairing catalytic demethylation, showed a much lesser impact. While germline Utx disruption arrests embryonic development at the time of organogenesis, this involves demethylase-independent activities of Utx, as shown by analyses of mutant mice expressing a catalytically inactive version of the protein (97–100). Germline disruption of Jmjd3, or disruption of Jmjd3 and Utx demethylase activity, are compatible with the development of most organs and systems, although it results in death of newborn mice due to the impaired development of the brain center controlling respiratory rhythm (101–103).

A tentative explanation for this apparent paradox is that “dilution” of H3K27Me3 marks at each cell division could make Jmjd3 and Utx demethylase, but not demethylase-independent, activities dispensable during differentiation processes associated with cell proliferation. In antigen-activated mature T cells, which extensively proliferate, such “dilution” could account for the limited effect of Utx disruption on H3K27Me3 distribution during the differentiation of follicular helper T cells (104). However, other observations challenge the idea that “dilution” can efficiently clear the mark. Jmjd3 disruption increased H3K27Me3 levels at more than 2,500 genes during the differentiation of Th1 effector CD4^+^ T cells (105), which is also accompanied by proliferation. Additionally, catalytic demethylation serves important functions *in vivo*, as it mediates in part the activity of Jmjd3 in macrophage effector differentiation (101) or in the development of the brain respiratory center (102), and of Utx in somatic cell reprogramming (106). As detailed below, studies of Jmjd3 and Utx functions in developing T cells shed light on this question.

### Role of H3 K27 Methyl Transferases and H3K27Me3 Demethylases During T Cell Development

Analyses of genomic H3K27Me3 deposition by chromatin immunoprecipitation followed by deep-sequencing (ChIPseq) suggested that this modification was important for transcriptomic changes during late αβ T cell differentiation (43, 44). Changes (increase or decrease) in H3K27 tri-methylation were detected at hundreds of promoters during the differentiation of DP into CD4 SP thymocytes (43, 44). Of specific interest were the almost complete removal of the mark at the genes encoding the CD4-differentiating transcription factor Thpok (38, 39), the S1pr1 receptor required for thymic egress (40), and the transcription factor Klf2, involved in the terminal maturation of SP thymocytes and S1pr1 expression (107). Conversely, increased H3K27Me3 decoration was observed at genes silenced during αβ T cell differentiation, including those encoding the recombinases Rag1 and Rag2. These changes in H3K27 methylation raised the possibility that mutations in Ezh1 and Ezh2 methyl transferases, or in Jmjd3 and Utx demethylases, would affect positive selection and the subsequent differentiation of αβ T cells in the thymus.

Experimental assessments of these predictions have produced mixed results. Deletion of Ezh2, the predominant H3K27 methyltransferase in the T cell lineage, has no reported impact on the differentiation of SP from DP thymocytes, unlike at earlier stages of T cell development, during the differentiation of iNK T cells, or in mature T cells (66–71, 108). This unexpected result does not imply that H3K27 methylation is not important for transcriptomic changes during the DP-SP transition, as the lack of an effect in DP thymocytes may reflect the potential functional overlap with Ezh1, highlighted in other developmental studies (64, 109–111) or the extended half-life of Ezh2 or H3K27Me3 molecules. A recent report pointed out to mechanisms controlling the stability of Ezh2 in activated T cells (112); future studies will address if it is controlled in developing thymocytes as well.

**Figure 3:**
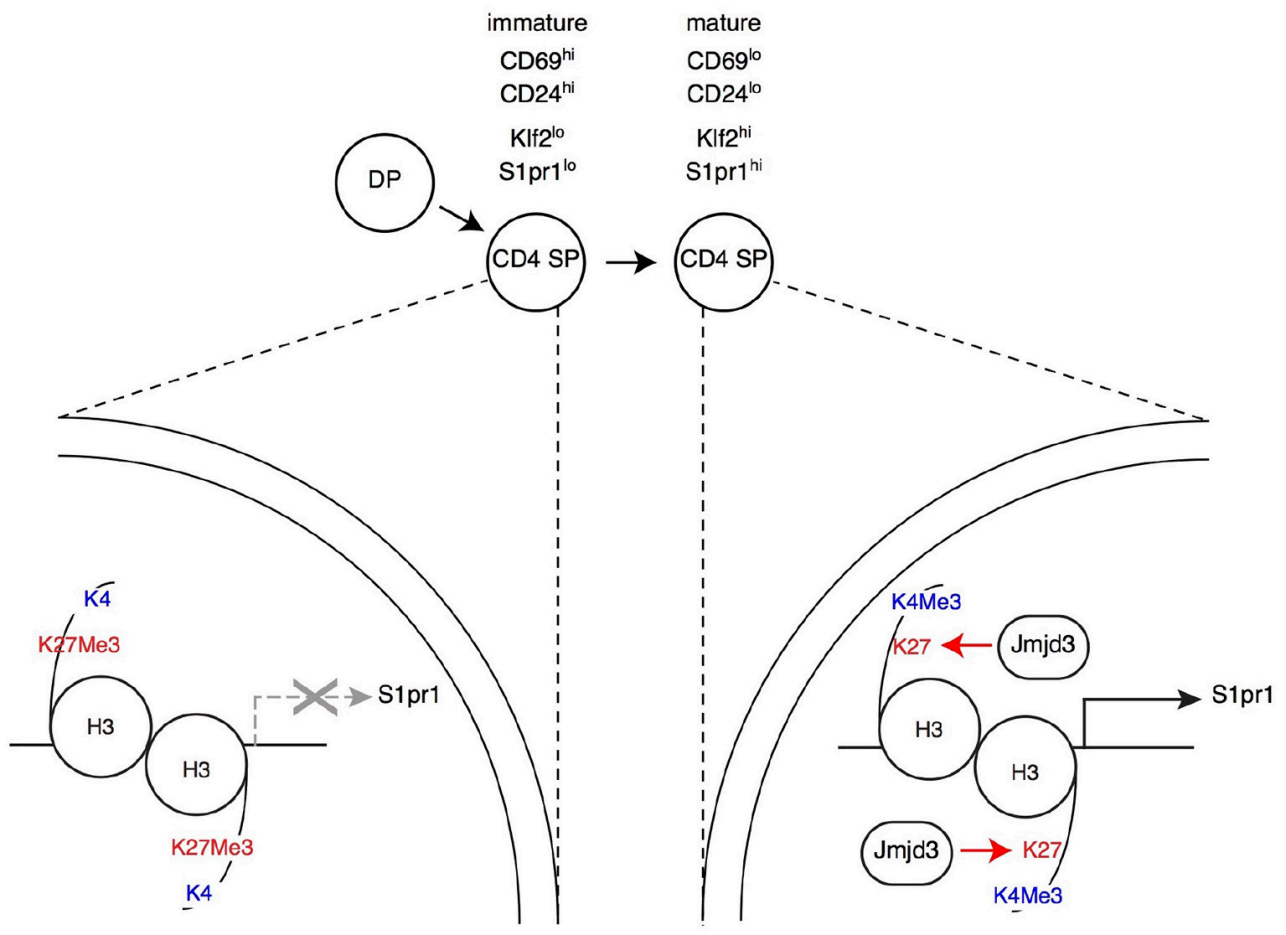
Impact of H3K27Me3 demethylation on late thymocyte differentiation Immature SP thymocytes (characterized by their expression of surface markers CD69 and CD24, as indicated) have low expression of the surface receptor S1pr1 (needed for thymic egress) and of the transcription factor Klf2 (needed for S1pr1 expression). Expression of both genes increases in mature SP thymocytes, allowing their export to the bloodstream and secondary lymphoid organs. In immature SP cells, the promoters of *S1pr1* and *Klf2* are enriched in the repressive H3K27Me3 mark, whereas the “active” H3K4Me3 mark is absent (**left**, depicted here for *S1pr1*). Thymocyte maturation is accompanied by an inversion of this pattern at both genes **(right)**. The H3K27Me3 demethylase Jmjd3 (with functional overlap with the related protein Utx, not depicted) is needed to “erase” the H3K27Me3 mark at *S1pr1*, for *S1pr1* expression and for thymic egress. Note that Jmjd3 is expressed at similar levels in both mature and immature SP cells (not shown in the latter for simplicity), suggesting that it is recruited to target genes through interactions with sequence-specific transcription factors.

The reciprocal experiment, namely deletion of Jmjd3 or Utx targeted to DP thymocytes, showed at first glance similar results as mice lacking either or both enzymes had CD4 and CD8 SP thymocytes and T cells (44, 101, 105). However, a detailed analysis showed that both enzymes are important for late T cell differentiation (44): Jmjd3 and Utx double-deficient mice had increased numbers of mature CD4 and CD8 SP thymocytes but reduced numbers of peripheral T cells; inactivation of either enzyme resulted in more limited effects, more pronounced for Jmjd3 than for Utx, consistent with functional overlap. Gene expression analyses and reconstitution experiments showed that these enzymes were needed for the expression of S1pr1, the sphingosine receptor required for thymic egress (40), and that this requirement accounted at least in part for their impact on late T cell differentiation (Figure 3) (44). Although the impact of Jmjd3 and Utx double-disruption on S1pr1 expression and T cell development was limited in animals expressing a diverse endogenous TCR repertoire, it resulted in an almost complete developmental block at the SP thymocyte stage in transgenic mice in which thymocytes all expressed a single TCR specificity, or when the development of mutant thymocytes was assessed in mixed bone marrow chimera, where they developed in competition with wild-type control cells. These findings indicated that loss of Jmjd3 and Utx activities can be compensated, in part, by changes in the repertoire of thymocytes completing their differentiation, and therefore suggested that H3K27Me3 demethylases contribute to gene expression in coordination with signals coming from TCR engagement.

Analyzing the impact of these enzymes on H3K27 methylation status and the transcriptome gave unexpected results. Even though DP and SP thymocytes are non-dividing cells, the inactivation of Jmjd3 and Utx had a highly specific impact on H3K27Me3 distribution (44). Unlike in a study of Jmjd3-deficient effector T cell differentiation (105), double-deficient thymocytes showed no general trend toward increased H3 K27 tri-methylation, whether at promoters or in non-promoter regions. Rather, H3K27Me3 density was significantly enhanced at fewer than 1% of loci (44), many of which were genes at which H3K27Me3 was normally removed during the DP to the CD4 SP transition, including *S1pr1* (Figure 3). This indicated a role of Jmjd3 and Utx in the dynamics of differentiation-induced H3K27Me3 erasing, rather than in its steady-state homeostasis. Intriguingly, deletion of Jmjd3 and Utx failed to affect H3K27Me3 erasing at a subset of promoters induced in differentiating αβ lineage thymocytes and at which H3K27Me3 is normally removed, including that of the gene encoding Thpok (44). The latter was in line with the lack of an effect of Jmjd3 and Utx on the differentiation of CD4 SP thymocytes and Thpok expression, and suggested that additional mechanisms contribute to H3K27Me3 removal. Similarly, the differentiation of MHC I-signaled thymocytes into the CD8^+^ was not affected by the double disruption of Jmjd3 and Utx (although the terminal maturation of CD8 SP cells was impaired to an extent similar to that of their CD4 SP counterparts).

Aside from *S1pr1*, the impact on the transcriptome of differentiating SP thymocytes was limited to a small number of genes, many of which were normally up-regulated during the terminal differentiation of SP thymocytes, including *Klf2* (44). Expression of most of these genes was reduced by the double disruption, suggesting that the impact of Jmjd3 and Utx on gene expression was mostly stimulating (in line with their “erasing” the repressive H3K27Me3 mark) (44).

### H3 K27 Methylation and iNK T Cell Development

Although the development of iNK and conventional T cells differs in important respects, both subsets differentiate from DP thymocytes upon engagement of their TCR by intrathymic ligands. Nonetheless, because of developmental steps unique to iNK T cells, disruption of H3 K27 methylation or demethylation has specific effects on their differentiation (summarized in Supplementary Table 1). Initial hints came from analyses of Ezh2-deficient thymocytes (70). Although it has no detectable effect on the development of conventional T cells, Ezh2 disruption in DP thymocytes results in increased numbers of iNK T cells, an effect particularly pronounced on IL-4-producing NKT2 cells and associated with increased PLZF expression.

Most remarkably, Ezh2 disruption “uncouples” iNK T cell differentiation from TCR specificity. Normally, PZLF expression and the acquisition of effector functions are characteristic of CD1d-restricted NK T cells, and of related “innate” T cells subsets restricted by non-classical MHC or MHC-like molecules, including mucosal-associated invariant T (MAIT) cells (113). Unexpectedly, Ezh2 deletion resulted in the appearance of large populations of T cells expressing PLZF, producing effector cytokines (including IL-4 and IFNγ), but without detectable binding to αGalCer-CD1d complexes and therefore distinct from type I iNK T cells (70). Additional lines of evidence supported the conclusions that these “NK T wannabe” are not type II NK T cells. Unlike type II NK T cells (50), they express a diverse TCR repertoire characteristic of conventional T cells, and they could develop in mice expressing an MHC II-restricted transgenic TCR specific for ovalbumin, which normally directs the differentiation of conventional CD4^+^ T cells. In line with their expression of PLZF, Ezh2-deficient NK T cell “wannabes” had no H3K27Me3 accumulation at the promoter of the gene encoding this factor, unlike conventional T cells (70). Thus, these experiments indicated that H3K27Me3 methylation restrains PLZF expression and effector differentiation to CD1d-restricted T cells and other subsets of innate T cells.

Studies of histone demethylase functions provided a mirror image of these findings. In contrast to their selective impact on late thymic maturation in conventional thymocytes, Utx and to a lesser extent Jmjd3 were found to be important for multiple aspects of iNK T cell development (13, 70, 114). Inactivation of both enzymes causes a broad block in the development of iNK T cells in the thymus, with a similar impact on liver iNK T populations. The block is contemporary with the up-regulation of PLZF and the acquisition of effector functions. However, there is no evidence that Utx is needed for PLZF up-regulation. Rather, it seems important to enforce the PLZF-mediated transcriptomic program characteristic of iNK T cell differentiation; consistent with this idea, Utx binds to PLZF molecules in iNK T cells (13). Of note, it is possible that additional mechanisms mediate the impact of Utx and Jmjd3 on iNK T cells, as the developmental block in Utx-deficient iNK T cells was more marked for T-bet-expressing and IFNγ-producing NKT1 cells than for the NKT2 and NKT17 subsets. Future studies will address these questions.

### Mechanistic Considerations

An important question raised by these observations is whether the impact of Jmjd3 and Utx on T cell development is mediated by their catalytic demethylase activity, since it is dispensable in embryonic development (97–100, 103). Multiple lines of evidence point to the importance of catalytic demethylation in developing T cells. Initial insight came from comparisons of female and male mice, because the gene encoding Utx (*Kdm6a*) is located on chromosome X. Accordingly, female cells carry (and express) two *Kdm6a* alleles; in contrast male cells express Utx from their single *Kdm6a* allele and the Y chromosome-located *Uty* gene, encoding the Utx-related protein Uty. Although lacking demethylase activity, Uty is functionally redundant with Utx during the development of male mice (103). In contrast, the impact of Jmjd3 and Utx disruption on conventional CD4 SP thymocyte maturation is the same in female and male cells (44). This indicates that demethylase-dead Uty is insufficient to promote thymocyte development, and therefore supports the idea that H3K27Me3 demethylase activity is required.

Three results from analyses in iNK T cells corroborate this conclusion. First, as in conventional thymocytes, Uty failed to rescue the defect caused by Utx disruption (114). Second, the combined deletion of Utx and Ezh2 resulted in a milder defect in iNK T differentiation, suggesting that the two proteins have opposite effects on a common target (114). Last, retroviral transduction “rescue” experiments directly demonstrated that a mutant of Utx lacking catalytic activity failed to restore iNK T cell differentiation from Utx-deficient thymocytes, unlike wild-type Utx (13).

Studies in thymocytes also raised the intriguing possibility that demethylase and demethylase-independent functions synergize for optimal gene expression. In mature conventional thymocytes, *S1pr1* gene expression depends both on H3K27Me3 demethylase activity (44) and on Ptip1 (115) an Utx-associated component of KTM2 complexes (87), suggesting that Utx could contribute to both functions. In differentiating iNK T cells, it was reported that Utx affects the chromatin accessibility of super-enhancers (chromosomal regions associating multiple enhancer elements and operationally defined by continuous high density stretches of H3K27Ac in ChIPseq experiments) and therefore presumably their activation (13). Indeed, Utx promoted expression of genes located near Utx-dependent super-enhancers. These results support the idea that Utx, through recruitment to gene regulatory regions by sequence-specific transcription factors (including PLZF in iNK T cells) contributes to enhancer activation.

## Concluding Remarks and Perspectives

The work summarized in this review highlights the importance of H3 K27 methylation in the development and function of T cells. Analyses of its function during cell differentiation face numerous challenges, including (i) the genome-wide deposition of the mark and its implied pleiotropic impact, (ii) the multiplicity of protein and protein complexes involved in the “writing,” “reading,” and “erasing” of the mark, with various degree of functional overlap, and (iii) the multifunctional nature of many components, and specifically H3K27Me3 demethylases. Nevertheless, studies over the past few years have brought important clarifications on the function of this mark in T cell development, both on its impact on the transcriptome of differentiating cells and its biological consequences, and on the mechanisms that underpin this impact.

Several important questions remain to be addressed. In particular, while it is clear that complete disruption of PRC1 activity (through inactivation of both Ezh1 and Ezh2, or of the non-redundant component Suz12) abrogates H3 K27 methylation and results in a major disruption of cell homeostasis and differentiation, the consequences of the double Jmjd3-Utx disruption are less striking, both on H3K27Me3 and developmental fates. At the gene level, evidence in non-dividing thymocytes that H3K27Me3 is “erased” despite Jmjd3 and Utx disruption (e.g., at the gene encoding Thpok) (44) indicates the involvement of additional mechanisms. While the involvement of other JmjC-family enzymes in H3K27Me3 demethylation cannot be excluded, there is little supporting evidence at present (74). Only Kdm4 family members have been reported to act on H3K27Me3 (116), and their actual activity remains to be clarified (117). Of note, the fact that Jmjd3 and Utx are required for H3K27Me3 clearance at other promoters (e.g., *S1pr1*) indicates that such effects would be gene specific. A distinct and tantalizing possibility is that, even in non-dividing cells, H3K27Me3 is functionally erased by nucleosome replacement rather than (or in addition to) catalytic demethylation. Replacement mechanisms (118) deposit nucleosomes containing the H3 variant H3.3 at actively transcribed genes (119–121) and could therefore “erase” the H3K27Me3 mark if such newly deposited nucleosomes contained un-methylated H3.3.

It will be important to integrate the dynamics of H3 K27 methylation in the broader context of epigenetic control of gene expression. Much progress has been made understanding the mutual relationships of activating and repressive histone marks. H3 K27 methylation and acetylation are biochemically mutually exclusive, and accordingly exert opposite effects on gene expression. More strikingly, evidence is accumulating that H3 K4 and K27 methylations, which are typically found in active vs. silent genes or enhancers, respectively, are the end products of enzymatic complexes that coordinate writing of one mark with erasure of the functionally opposite mark. That is, Ktm2/MLL complexes associate both an H3 K4 methyl transferase and H3K27Me3 demethylases, whereas PRC2 complexes associate H3 K27 methyl transferase activity and H3K4Me3 demethylases of the Jarid1-RBP2-Kdm5 family (62, 122, 123).

How these activities integrate with the other key histone repressive mark, H3 K9 methylation, has been addressed in various experimental systems (124) but remains to be explored in T cells. While H3K9Me3 has been traditionally associated with constitutive heterochromatin, there is ample evidence that H3 K9 methyl transferases contribute to the control of lineage-specific gene expression, including those involved in T cell development and function (125–128). Additional data suggest that PRC2 and H3 K9 methyl transferase complexes could share components, including Jarid2 (or Jumonji, the founding member of the JmjC family), which was shown to restrain PLZF expression in and iNK T differentiation of thymocytes and to promote H3 K9 but not K27 trimethylation at the promoter of the gene encoding PLZF (129).

Last, histone modifications are super-imposed on the dynamics of DNA methylation, which was the first epigenetic modifications identified in developing T cells at the *Cd4* and *Cd8* loci. T cell development is accompanied by reduced methylation at CpG islands in both loci following commitment to the αβ lineage and onset of CD4 and CD8 expression, followed by partial, lineage specific, remethylation of the silenced coreceptor gene (130). More recent studies have pointed to the importance of DNA methylation in the maintenance of *Cd4* silencing in mature CD8^+^ T cells, suggesting a yet to be determined coupling between the mechanisms writing the methyl mark (presumably involving DNA methyl transferases Dnmt3 isoforms) and those ensuring the active repression of *Cd4* in CD8-differentiating thymocytes (131).

Conversely, work in the past few years has identified a complex mechanism erasing cytosine methylation, without actual catalytic demethylation, initiated by oxidization of methyl cytosine catalyzed by Tet1, Tet2, and Tet3 enzymes (of the ten-eleven-translocation family) (132, 133). Although the full impact of Tet enzymes on the development of conventional αβ T cells remains to be elucidated, they are essential to restrain the activation of iNK T cells (134, 135). While the current evidence indicates an impact on cell proliferation, deletion of Tet enzymes also impaired the differentiation of NKT1 cells, suggesting an additional impact on cell differentiation. Thus, it will be important to understand the respective contributions of DNA methylation and H3 K27 trimethylation in the control of T cell homeostasis and function, especially in the light of studies suggesting that DNA methylation antagonizes H3K27Me3 deposition (124).

## Author Contributions

The author confirms being the sole contributor of this work and has approved it for publication.

### Conflict of Interest Statement

The author declares that the research was conducted in the absence of any commercial or financial relationships that could be construed as a potential conflict of interest.
