# Supplementary material for: Control of Intra-Thymic αβ T Cell Selection and Maturation by H3K27 Methylation and Demethylation
Source: Front Immunol. 2019 Apr 3;10:688. doi: 10.3389/fimmu.2019.00688 (PMC6456692; doi:10.3389/fimmu.2019.00688)
Supplement: Supplementary file 1 [file Table_1.docx]

**Supplementary Table 1: Summary of H3K27Me3 modifier functions in T cell development**

| Jmjd3 | T Acute lymphoblastic leukemia development | Ntziachristos et al. (136) |
| --- | --- | --- |
| Utx | T Acute lymphoblastic leukemia suppression | Ntziachristos et al. (136) |
|  | Follicular Helper T cell differentiation | Cook et al. (104) |
| Redundant functions of Jmjd3 and Utx | INK T cell development | Dobenecker et al. (70); Beyaz et al. (13); Northrup et al. (114) |
|  | T cell effector differentiation | Miller et al. (89); Li et al.(105); Liu et al. (137) |
|  | Late thymocyte development | Callen et al.(115); Manna et al. (44) |
| Polycomb complex components | Early hematopoiesis | Lee et al. (95); Mochizuki-Kashio et al. (111) |
|  | Early T cell development | Su et al. (66); Miyazaki et al. (67) |
|  | CD8 T cell memory | Zhang et al. (69); Gray et al. (71); He et al. (108) |
|  | T cell effector differentiation | Tumes et al. (68); Zhang et al. (69) |
| Ezh2 | INK T cell development | Dobenecker et al. (70); Vasanthakumar et al. (84) |
|  | T cell receptor signaling | Su et al. (66); Dobenecker et al.(72) |
|  | Hematopoietic malignancy suppression | Mochizuki-Kashio et al. (111) |
